# Validation of a Visual Analogue Scale to measure the subjective perception of orgasmic intensity in females: The Orgasmometer-F

**DOI:** 10.1371/journal.pone.0202076

**Published:** 2018-08-29

**Authors:** Daniele Mollaioli, Stefania Di Sante, Erika Limoncin, Giacomo Ciocca, Giovanni Luca Gravina, Elisa Maseroli, Egidia Fanni, Linda Vignozzi, Mario Maggi, Andrea Lenzi, Emmanuele A. Jannini

**Affiliations:** 1 Department of Systems Medicine, University of Rome Tor Vergata, Rome, Italy; 2 Division of Endocrinology, Department of Experimental Medicine, Sapienza University of Rome, Rome, Italy; 3 Department of Biotechnological and Applied Clinical Sciences, University of L’Aquila, L’Aquila, Italy; 4 Department of Experimental and Clinical Biomedical Sciences, University of Florence, Florence, Italy; University of Insubria, ITALY

## Abstract

The female orgasm represents one of the most complex functions in the field of human sexuality.

The conjunction of the anatomical, physiological, psycho-relational and socio-cultural components contributes to make the female orgasm still partly unclear. The female orgasmic experience, its correlates and the relation with sexual desire, arousal and lubrication as predictors are highly debated in scientific community. In this context, little is known about the impact of female sexual dysfunction (SD) on sexual pleasure expressed by subjective orgasmic intensity, and there are no suitable psychometric tools suited to investigate this dimension. Thus, we validate, in female subjects, a Visual Analogue Scale (VAS) that we named Orgasmometer-F, to verify if SD is accompanied by a lower perceived orgasmic intensity. A total of 526 women, recruited through a web-based platform and from sexological outpatient clinic, were enrolled in the study. They were divided into, on the basis of the Female Sexual Function Index (FSFI) score in two groups: 1) 112women suffering from SD, (SD Group); and 2) 414 sexually healthy women (Control Group). The participants were requested to fill out the Orgasmometer-F, recording orgasmic intensity on a Likert scale from 0 (absence of orgasmic intensity) to 10 (maximum orgasmic intensity experienced). Women with SD experienced significantly lower orgasmic intensity than controls, as measured by the Orgasmometer-F (*p < 0*.*0001*). Interestingly, masturbatory frequency was positively correlated with orgasmic intensity, as were the lubrication, orgasm and sexual satisfaction domains of the FSFI. The Orgasmometer-F was well understood, had a good test-retest reliability (ICC = 0.93) and a high AUC in differentiating between women with and without sexual dysfunction (AUC = 0.9; *p < 0*.*0001*). The ROC curve analysis showed that a cut-off <5 had 86.5% sensitivity (95% CI 82,8–89,6), 80.4% specificity (95% CI 71.8–87.3), 75.4% positive predictive value (PPV) and 89.5% negative predictive value (NPV). In conclusion, the Orgasmometer-F, a new psychometrically sound tool for measuring orgasmic intensity in female population, demonstrated that SD impair orgasmic intensity.

## Introduction

Female orgasm is a neuromuscular phenomenon triggered by sexual (somatic and mental) stimuli, accompanied by anatomical and physiological responses including vasocongestion of the erectile tissues, lubrication, and pelvic contractions that induce intense pleasurable sensations [1–5]. This female orgasm is not yet fully understood and defined, because of the great variability in factors including localization [6–8], stimulation techniques [9,10], self-image [11–13] and quality of romantic relationship [10,14]. It is therefore very difficult to describe female orgasm simply and concisely. For this reason, the analysis of orgasmic experience in women is not yet complete and merits further investigation of necessary details.

The mechanisms of interaction between the external and internal clitoris and the anatomical structures that contribute to the formation of the orgasmic platform have been described [1]. However, there is also a larger area involved in sexual stimulation, called the clitourethrovaginal (CUV) complex [15–17], a variable, multifaceted morpho-functional area that, when properly stimulated during penetration, could induce orgasmic response.

In addition, orgasmic function is strictly related with the other sexual response phases (desire, arousal, lubrication). The capability of reaching a satisfying orgasm needs the ability of having sexual fantasies and partially loosing cognitive control, making the arousal increase and having an efficient lubrication during all the sexual experience. [18,19]

In contrast, little is known about the orgasmic experience, meaning the feelings and cognitions experienced during orgasm. Terms like orgasmic intensity, pleasure and satisfaction should be included in the evaluation of orgasmic experience [20] and related to the different ways of achieving orgasm [21], to its cognitive-affective aspects [22], and to satisfaction with relationship itself [14]. Despite its importance, this aspect is entirely neglected in the investigation of sexual dysfunction, even though for many women orgasm is the ultimate goal of intercourse [23] and a source of sexual satisfaction with or without their partner [10].

A negative or absent orgasmic experience is often related to a general condition of sexual dysfunction [24]. The last Diagnostic and Statistical Manual of Mental Disorders (DSM5) includes in the “*Female Orgasmic Disorder"* (FOD) both the absence of orgasm (anorgasmia) and the delayed or reduced intensity of orgasm [25]. The female sexual dysfunctions (SD) classified according to medical and psychiatric taxonomies [26,27] induce in the couple a sexual discomfort that affects, to varying degrees, all phases of the sexual response cycle. In fact, a negative orgasmic experience is often both the cause and consequence of difficulties in relation to hypoactive sexual desire disorder, vaginal dryness and inadequate arousal [24,27].

Despite the current debate on female orgasm, the importance of orgasm itself in the couple’s health and the possible impact of reduced or absent orgasmic experience in provoking or amplifying female SD, within the several psychosexological questionnaires mentioning the orgasmic function [28–33], none specifically measures the female orgasmic intensity. Previous attempts to assess female orgasm have been focused in assessing the phenomenological sensations (sensory and cognitive-affective), with a two-dimensional model, associated with orgasm (Orgasm Rating Scale) [20] or attempting to capture the specific bodily sensations that are associated with climax (Bodily Sensations of Orgasm questionnaire) [34]. Conversely, a well-validated Visual Analogue Scale (VAS) and named the “Orgasmometer”, is currently available in the clinical andrology to assess, with excellent psychometric qualities, exclusively the intensity of orgasm in male [35].

Thus, the aim of this study was to establish and validate a new psychometric tool, the Orgasmometer-F measuring the orgasmic intensity in a female population with SD.

## Material and methods

### Study population

A consecutive series of 643 and 35 subjects were enrolled, respectively, through a web-based platform ad hoc build and publicized by social media, or from our sexual medicine outpatient clinic. All the subjects were invited to fill out into the web-based platform a sociodemographic questionnaire exploring clinical and sexual history, the Female Sexual Function Index (FSFI) questionnaire and the Orgasmometer-F (see later).

**Fig 1:**
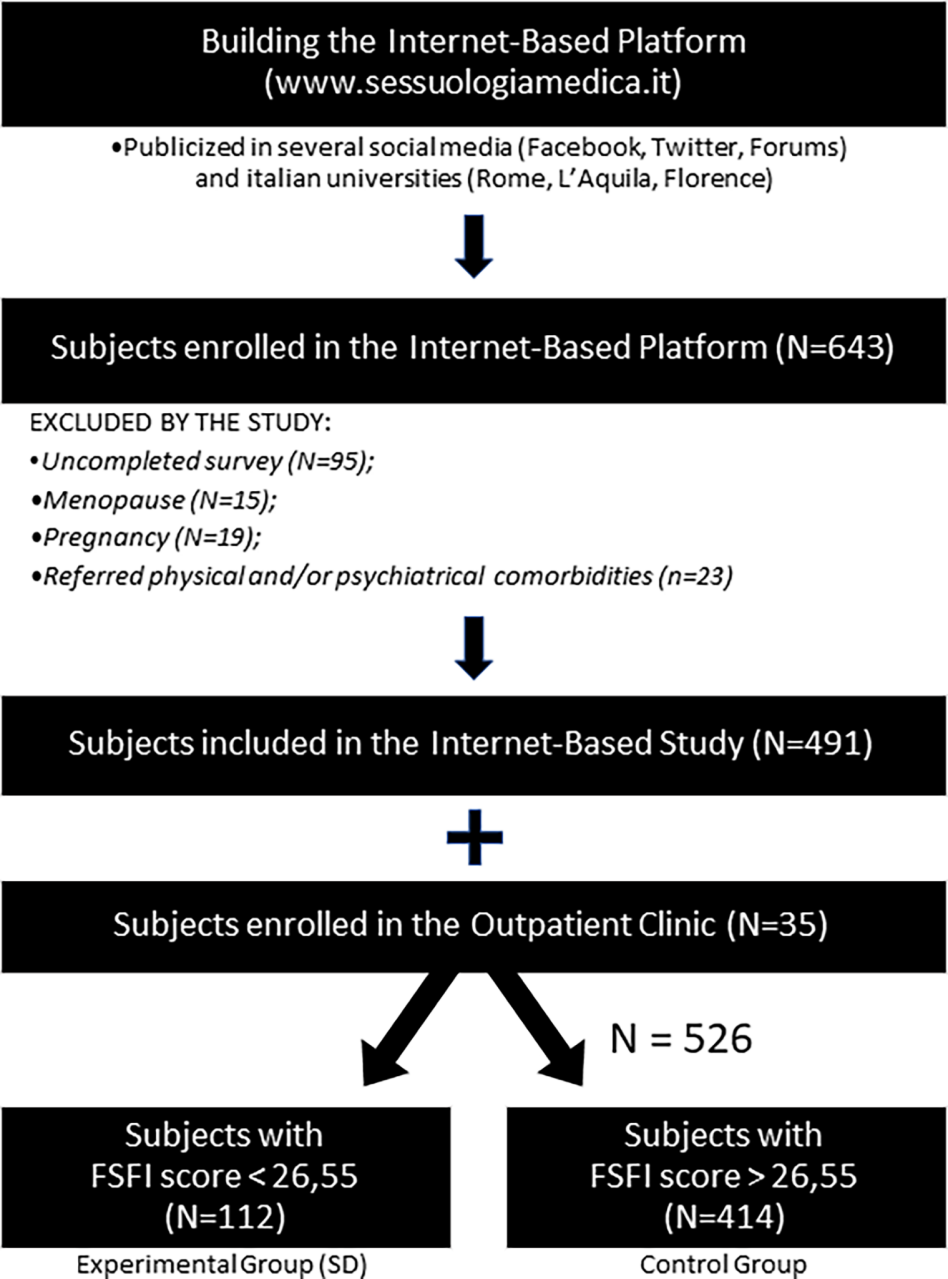
A flowchart showing the study design.

Based on the presence or absence of reported SD (evaluated by the clinical cut-off of the FSFI questionnaire, i.e.< 26,55) [29], subjects were divided into two groups. The **SD Group** with pathological FSFI score was composed of 77 women enrolled online (OL-SD subgroup) and 35 women recruited as outpatients with an SD (OP-SD subgroup) and the **control group** of 414 women with normal FSFI filled out in the web-based platform. The remaining 152 subjects from the web-based platform were excluded from the study on the basis of the exclusion criteria (see below), irrespectively of the FSFI score. (Fig 1)

Among the inclusion criteria, women during the six months preceding the enrollment had to be sexually active and had to have experienced orgasmic pleasure (clitorally and/or vaginally activated [7]). The exclusion criteria (evaluated by the sociodemographic questionnaire) for both groups were as follows: age below 18; menopause or pregnancy; referred presence in women or in their partners of medical conditions influencing orgasmic experience (multiple sclerosis, diabetic nerve damage, spinal cord injury, hormonal disorder, menopause, chronic pelvic pain, and endometriosis) or psychiatric diseases; use of psychiatric medications affecting orgasmic intensity (hypnotics, anxiolytics, antidepressants, antipsychotics); drug use (alpha-sympathetic drugs, opioids, cocaine); absence of orgasmic experience during the last 6 months.

All subjects were asked to inspect all the study information, to give their written informed consent to the use of their personal information and to complete all the questionnaires.

All subjects participated voluntarily. The “Azienda Policlinico Umberto I” Ethics Committee approved the study protocol.

### Main outcome measures

#### The female sexual function index

This standardized psychometric questionnaire is a validated tool to evaluate the presence of sexual dysfunction [29,30], which has been validated also in Italian language [36]. It has 19item in six domains exploring overall female sexual function, based on the DSM IV-TR criteria [37]. It takes about 15 minutes to complete and the response options for each item are on a 5-6-points Likert scale. Scores below the clinical cut-off point (26.55) indicate the presence of sexual dysfunction in the previous 4 weeks [29].

#### The Orgasmometer-F

**Fig 2:**
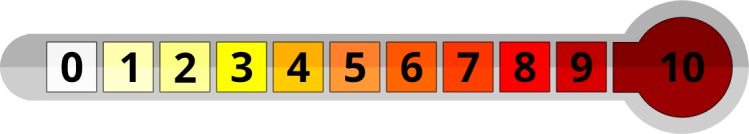
The Orgasmometer-F. Considering a Likert scale ranging from 0 to 10, where 0 corresponds to the absence of orgasmic perception and 10 to maximum perceived orgasmic intensity, how do you evaluate your orgasmic intensity in the last six months?

The Orgasmometer-F is a psychometric tool evaluating the subjective perception of orgasmic intensity (Fig 2). It is structurally based on the Visual Analogue Scale (VAS) [38], a psychometric tool for the evaluation of subjective perception of pain intensity. It was recently validated in men [35]. Orgasmic intensity is reported through both a numeric scale and chromatic gradation, ranging from 0 (white), corresponding to the absence of orgasmic intensity, to 10 (deep red), corresponding to the highest level of orgasmic intensity. Unlike in men, where orgasmic intensity was evaluated for the four weeks preceding test administration, we chosen a 6-months period in order to adhere to DSM criteria of FOD regarding the reduction of orgasmic intensity.

### Statistical analysis

In order to verify the distribution (normal or non-normal) of the variables, the Kolmogorov-Smirnov test has been operated for each study variable. Since continuous variables were not normally distributed, Mann-Whitney test for the comparison of independent samples and Spearman’s Rho for the correlation have been performed with a normal distribution were expressed as mean ± standard deviation, whereas non-normally distributed continuous variables were expressed as median (95% CI). Frequencies and percentages were computed for dichotomous variables. Differences in categorical variables were tested for statistical significance with the Chi-squared test. [39]

Orgasmic intensity scores in the study groups [OL-SD vs. OP-SD and SD group vs. control group] were compared by multivariate analysis of covariance (MANCOVA) was performed using relationship status, masturbatory frequency and FSFI domains (desire, arousal, lubrication, orgasm, pain, satisfaction) as covariates. A stepwise multiple regression was used to identify significant determinants of perceived orgasmic intensity.

To compare the predictive ability of the Orgasmometer-F in females with and without sexual dysfunction, receiver-operated characteristic (ROC) curves were used to determine the cut-offs that best discriminated between the individuals with high and low levels of perceived orgasmic intensity. To evaluate specificity and sensitivity, ROC analyses were performed using the method recommended by DeLong et al. [40]. A p value of ≤0.05 was considered as statistically significant for each statistical analysis.

## Results

To evaluate reliability, intraclass correlation coefficient (ICC) analysis was performed on Orgasmometer-F repeated measures, collected on day 0 and 14 (n = 35). The ICC was 0.93 (95% CI 0.91–0.95), showing a high test-retest reliability.

As the SD group consisted of two subgroups (OL-SD and OP-SD groups), a MANCOVA analysis adjusted for relationship status, masturbatory frequency and FSFI domains (desire, arousal, lubrication, orgasm, pain, satisfaction) was performed to assess whether they differed in their Orgasmometer-F score, but found no difference (OL-SD: mean: 5.25; 95% CI 4.93–5.58; OP-SD: mean 5.05; 95% CI 4.55–5.56; p = 0.519). The two subgroups were therefore unified and considered as a single SD group.

The socio-demographic and clinical variables of the sample are shown in Table 1. Clinical and demographic variables differ significantly between the SD and control groups, except for age and BMI variables.

**Table 1:** Sociodemographic and clinical characteristics.

	SD Group N = 112	Control Group N = 414	p
Age^a^	26 (25–27)	26 (25–26)	0.0554^**§**^
BMI^a^	21.2 (20.2–22.1)	22.2 (21.7–22.6)	0.1104^**§**^
University Degree^b^	52.7 (59)	66.2 (274)	0.0117*
In a relationship^b^	67.9 (76)	79.7 (330)	0.0116*
Masturbatory frequency ^b^ (>once a week)	50.9 (57)	62.8 (258)	0.0295*
***FSFI Subscales Scores***			
Desire^a^	3.60 (2.4–3.6)	4.80 (3.6–4.8)	**<0.0001**^**§**^
Arousal^a^	3.60 (3.0–4.2)	5.40 (4.8–5.7)	**<0.0001**^**§**^
Lubrication^a^	4.20 (3.6–4.8)	6.00 (5.4–6.0)	**<0.0001**^**§**^
Orgasm^a^	3.60 (2.8–4.0)	5.60 (4.8–6.0)	**<0.0001**^**§**^
Pain^a^	4.00 (2.4–5.2)	5.60 (4.8–6.0)	**<0.0001**^**§**^
Satisfaction^a^	3.60 (2.8–4.8)	5.60 (4.8–6.0)	**<0.0001**^**§**^
Total Score^a^	22.6 (20.9–24.2)	31.7 (29.9–33.6)	**<0.0001**^**§**^

^a^ Median (Interquartile Range)

^b^ % yes (N)

^§^ Mann–Whitney test

* χ^2^ test

A MANCOVA analysis with relationship status, masturbatory frequency and FSFI domains (desire, arousal, lubrication, orgasm, pain, satisfaction) as covariates were therefore performed to assess any differences in the subjective perception of orgasmic intensity between the two groups. The SD group reported lower scores (mean 5.24; 95% CI 4.79–5.69) than the controls (mean 6.71; 95% CI 6.54–6.88; Table 2); this difference was statistically significant (p<0.0001).

**Table 2:** MANCOVA analysis for SD group and control group Orgasmometer-F values adjusted for covariates.

**Levene’s test for equality of error variances**					
*F*	*d*.*f*.^*a*^ *1*	*d*.*f* ^*a*^ *2*		*p*	
0,02432	1	524		0,876	
**Tests of Between-Subjects Effects**					
***Covariates***	***Sum of Squares***	***d*.*f*.**^*a*^	***Mean Square***	***F***	***P***
University Degree	0,531	1	0,531	0,267	0,606
In a relationship	5,394	1	5,394	2,713	0,100
Masturbatory frequency (>once a week)	18,771	1	18,771	9,443	**0,002**
*FSFI Subscales*					
Desire	0,767	1	0,767	0,386	0,535
Arousal	0,162	1	0,162	0,082	0,775
Lubrication	47,989	1	47,989	24,140	**<0,001**
Orgasm	24,697	1	24,697	12,424	**<0,001**
Pain	0,652	1	0,652	0,328	0,567
Satisfaction	14,013	1	14,013	7,049	**0,008**
**Coefficient of determination R**^**2**^		**0,4490**			
**R**^**2**^**-adjusted**		**0,4383**			
**Estimated Marginal Means**					
**Study groups**	**N**	**Mean**	**Std. Error**	**95% CI **^**b**^	
SD Group	112	5,2461	0,2284	4,7974 to 5,6948	
Control Group	414	6,7112	0,0856	6,5431 to 6,8793	
**Post hoc pairwise comparisons**					
**Study groups**	**Mean Diff**		**Std. Error**	**P **^**b**^	
FSD Group vs Control Group	- 1,4651		0,2795	*<0*,*0001*	

a Degrees of Freedom

b Bonferroni corrected

Despite significative differences between the two groups in being in a relationship and having an university education, these variables do not impact on the subjective orgasmic intensity, as well for sexual desire, arousal and coital pain domains of FSFI.

As masturbatory frequency, lubrication, orgasmic function and sexual satisfaction were found to be correlated with orgasmic intensity, a stepwise multiple regression analysis was then performed to investigate their relationship with perceived orgasmic intensity. This analysis suggested:

- a positive correlation between the lubrication FSFI domain score (r_partial_ = 0.318; p <0.0001) and the Orgasmometer-F score;
- a positive correlation between the orgasmic FSFI domain score (r_partial_ = 0.292; p <0.0001) and the Orgasmometer-F score;
- a positive correlation between the sexual satisfaction FSFI domain score (r_partial_ = 0.244; p <0.0001) and the Orgasmometer-F score;
- a positive correlation between the frequency of masturbation (r_partial_ = 0.137; p = 0.0017) and the Orgasmometer-F score.

Hence, lower scores in lubrication, orgasm and sexual satisfaction, as well as lower masturbation frequency, were associated with a lower subjective perceived orgasmic intensity.

**Fig 3:**
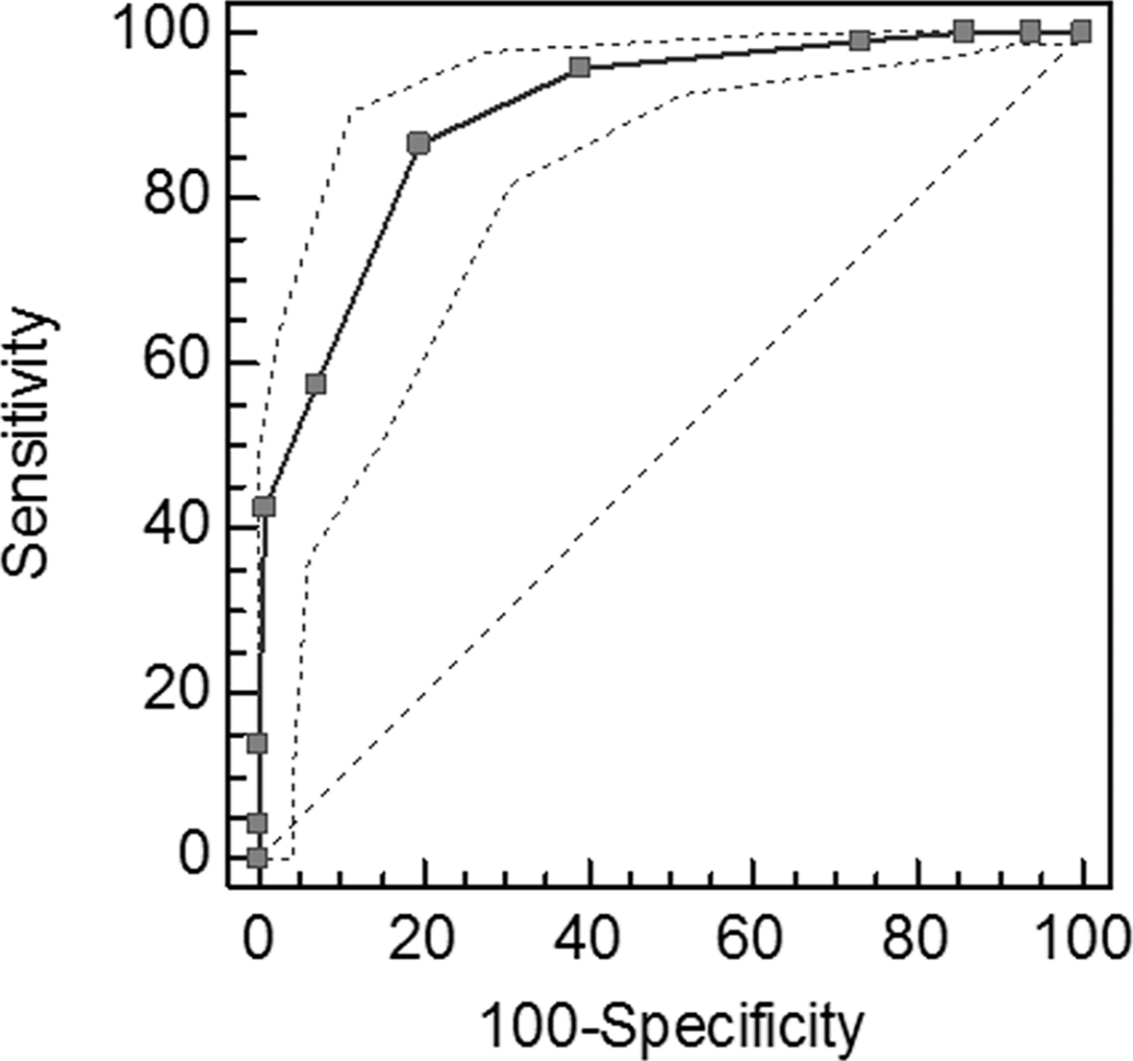
ROC curve analysis. The analysis was carried out to establish the Orgasmometer-F’s predictive ability to measure the subjective perception of orgasmic intensity.

Fig 3 shows the values of the ROC curve for the Orgasmometer-F. The perceived orgasmic intensity was evaluated by the following sentence: "*Considering a Likert scale ranging from 0 to 10*, *where 0 corresponds to the absence of orgasmic perception and 10 to maximum perceived orgasmic intensity*, *how do you evaluate your orgasmic intensity in the last six months*?". For the dichotomous classification variable, the SD group and control groups were coded as 1 or 0 respectively. The AUC was 0.9 (95% CI 0.871 to 0.924; p <0.0001).

Finally, to assess the Orgasmometer-F’s ability to measure low perceived orgasmic intensity, a sensitivity analysis was conducted. A cut-off of<5 was the optimal criterion for differentiating between a low and a high orgasmic intensity. At this cut-off, the sensitivity was 86.5% (95% CI 82,8–89,6), specificity is 80.4% (95% CI 71.8–87.3), Positive Predicted Value (PPV) was 75.4% and Negative Predictive Value (NPV) was 89.5%.

## Discussion

There are currently no psychometric tools that specifically measure the intensity of orgasm. For this reason, the aim of this study was to validate the Orgasmometer-F in the female population in order to identify situations in which women with SD might perceive low orgasmic sensations.

Overall, the Orgasmometer-F is a quick, easy tool for evaluating orgasmic intensity in women. It has a high test-retest reliability and high sensitivity, specificity, PPV and NPV. This single-item questionnaire could be used in clinical research to identify orgasmic difficulties related to other sexual dysfunctions and as an additional tool for the FOD diagnosis. In fact, the DSM5 criterion [24] "markedly reduced intensity of orgasmic sensations" in the diagnosis of FOD is evaluated by the personal assessment of the clinician. This study is, to our knowledge, the first to attempt to suggest how the Orgasmometer-F should be used in evaluating female orgasmic disorder.

The usefulness of the Orgasmometer-F in the female population is further supported by the fourth consensus of the International Consultation on Sexual Medicine (ICSM) [41], which proposed a new sexual dysfunction called *Hypohedonic Orgasm* and defined as “*lifelong or acquired decreased or low level of sexual pleasure with orgasm*” [41].

The primary endpoint of this study was to determine whether women with sexual dysfunction might perceive diminished orgasmic intensity compared to sexually healthy women. Our data provide quantitative evidence to support this idea.

The low confidence interval of the results obtained with the Orgasmometer-F for the control group of sexually healthy women has important implications. It might be explained by the nature of the instrument: the Orgasmometer-F evaluates orgasmic experience during a six-month period, unlike other questionnaires that limit their investigation to four weeks. This longer time means that women are likely to consider a varying level of pleasurable sensation and sexual satisfaction in answering the questionnaire [10,27]. Conversely, women with sexual dysfunction will report their discomfort in their experience of orgasm [10,24,27]. Moreover, since the female orgasm is a complex product of physical, emotional, cognitive and relational factors, it is reasonable to suppose that the “best” orgasm in women is yet to come. Factors such as anatomy [7,8,16,17,42–44], hormonal levels [45], age and sexual experience [46], self-awareness [11,47,48], sexual autonomy (i.e. the extent to which one feels that one’s sexual behaviors are self-determined) [49], ability to lose control during sexual activity [50] and partner-related sexual dysfunctions [51] are closely linked with orgasmic function. However, lacking so far a specific and dedicated psychometric tool, all these studies are not showing qualitative data on female orgasm.

Interestingly, although relational aspects are considered pivotal in the female sexual experience, not being in relationship does not impact on orgasmic experience. In fact, women with orgasmic difficulties tend to approach negatively both in autoerotism and partnered sex. [52,53] Among the factors that negatively affect orgasmic intensity, we identified low scores in the FSFI obvious domains of orgasm and sexual satisfaction but also in the domain of lubrication. This finding fully agrees with the idea that a SD in women is seldom restricted to just one of the phase of sexual response [26,27]. In fact, reduced lubrication can lead women to have difficulty reaching orgasm, to feel it less intensely and in the long term to judge their sexual relationship to be problematic and unsatisfactory. On the other hand, an efficient lubrication leads a woman to focusing better on sexual experience increasing, in a virtuous circle, both sexual desire and arousal [54] and having more probabilities to reach a pleasurable orgasm and feel itself sexually satisfied. In a recent study, over half of women who have difficulty reaching orgasm reported a SD, with greater difficulty in reaching adequate arousal / lubrication, have less sexual desire [54] and longer orgasmic latency times than sexually healthy women. In addition, these women reported less satisfaction in their sexual relationship [27].

Another factor that increases the orgasmic intensity is the adequate masturbatory frequency, quantified as one or more times a week. Autoeroticism in women appears to be associated with a wider repertoire of sexual fantasies and practices, as well as greater ease in reaching arousal and orgasm [55]. Conversely, feelings like shame and sense of guilt about masturbating were found in women with sexual difficulties [55]. Masturbation is a positive component in the structuring of female sexuality and genital sensations, increasing satisfaction in sexual intercourse with partners [48,56]. The findings of the present study are thus consistent with previous evidences, further highlighting the importance of autoerotic experience in sexual self-knowledge.

These findings, which are based on a subjective perception of orgasm, could nevertheless be reinforced with a future comparison of Orgasmometer-F values and objective measures, such as photoplethysmography [57], functional magnetic resonance imaging [58], or with pudendal somatosensory evoked potentials [59].

## Limitations

Several limitations in the present study should be noted. The first limitation is its cross-sectional design and lack of hormone testing. However, we are currently considering the effect of reproductive factors, such as menstrual cycle, pregnancy, and puerperium, associated with the intensity of female climax as measured by the Orgasmometer-F.

Furthermore, sexual fantasies were not investigated in this study, which may contribute to a better comprehension of the subjective orgasmic experience in females. We are currently including the investigation of sexual fantasies in the evaluation of orgasmic experience, both in males and females.

Convergent validity was not performed in this study. This is clearly due to a lack of another specific tool in literature to assess orgasmic intensity. Therefore, further investigations could be necessary to verify this aspect.

Lastly, the Internet-based enrollment of subjects presents some selection biases [60,61]. However, since sexuality itself typically represent a research field that can induce embarrassment in the participants, its investigation with Internet studies may reduce these possible negative effects [35, 62–66]. Moreover, the main inclusion criteria were the sexual activity, the experience of the orgasm and the ability to fill the FSFI, data that can be easily obtained both with a vis-à-vis interview and with an internet-based questionnaire. Finally, the use of a questionnaire, such as the FSFI, originally validated for auto-administration [29, 67], may mitigate this enrollment bias.

## Conclusions

In conclusion, this study demonstrated that female SD is associated with a lower perceived orgasmic intensity. Conversely, some important components of female sexuality such as lubrication, orgasm satisfaction and masturbation, have a positive correlation with perceived orgasmic intensity. The Orgasmometer-F was thus found to be a quick and simple tool for the assessment of the orgasmic experience in the female population.
